# A High Redox Potential Laccase from *Pycnoporus sanguineus* RP15: Potential Application for Dye Decolorization

**DOI:** 10.3390/ijms17050672

**Published:** 2016-05-05

**Authors:** Ana L. R. L. Zimbardi, Priscila F. Camargo, Sibeli Carli, Sidney Aquino Neto, Luana P. Meleiro, Jose C. Rosa, Adalgisa R. De Andrade, João A. Jorge, Rosa P. M. Furriel

**Affiliations:** 1 Department of Chemistry, Faculty of Philosophy, Sciences and Languages of Ribeirão Preto, University of São Paulo, Bandeirantes Avenue, 3900, Ribeirão Preto, SP 14040-901, Brazil; luccilatorre@usp.br (A.L.R.L.Z.); priscila@lcp.inpe.br (P.F.C.); sibelicarli@usp.br (S.C.); netaoaquino@gmail.com (S.A.N.); luanapm@usp.br (L.P.M.); ardandra@ffclrp.usp.br (A.R.D.A.); 2 Department of Molecular and Cell Biology and Protein Chemistry Center, CTC-Center for Cell Therapy-CEPID-FAPESP-Hemocentro de Ribeirão Preto, Ribeirão Preto Medical School, University of São Paulo, Bandeirantes Avenue, 3900, Ribeirão Preto, SP 14040-901, Brazil; jcrosa@fmrp.usp.br; 3 Department of Biology, Faculty of Philosophy, Sciences and Languages of Ribeirão Preto, University of São Paulo, Bandeirantes Avenue, 3900, Ribeirão Preto, SP 14040-901, Brazil; joajorge@ffclrp.usp.br

**Keywords:** *Pycnoporus sanguineus*, solid state fermentation, response surface methodology, laccase, purification, characterization, decolorization, remazol brilliant blue R, Reactive blue 4, bromophenol blue

## Abstract

Laccase production by *Pycnoporus sanguineus* RP15 grown in wheat bran and corncob under solid-state fermentation was optimized by response surface methodology using a Central Composite Rotational Design. A laccase (Lacps1) was purified and characterized and the potential of the pure Lacps1 and the crude culture extract for synthetic dye decolorization was evaluated. At optimal conditions (eight days, 26 °C, 18% (*w*/*w*) milled corncob, 0.8% (*w*/*w*) NH_4_Cl and 50 mmol·L^−1^ CuSO_4_, initial moisture 4.1 mL·g^−1^), the laccase activity reached 138.6 ± 13.2 U·g^−1^. Lacps1 was a monomeric glycoprotein (67 kDa, 24% carbohydrate). Optimum pH and temperature for the oxidation of 2,2’-azino-*bis*(3-ethylbenzthiazoline-6-sulfonate) (ABTS) were 4.4 and 74.4 °C, respectively. Lacps1 was stable at pH 3.0–8.0, and after two hours at 55–60 °C, presenting high redox potential (0.747 V *vs.* NHE). ABTS was oxidized with an apparent affinity constant of 147.0 ± 6.4 μmol·L^−1^, maximum velocity of 413.4 ± 21.2 U·mg^−1^ and catalytic efficiency of 3140.1 ± 149.6 L·mmol^−1^·s^−1^. The maximum decolorization percentages of bromophenol blue (BPB), remazol brilliant blue R and reactive blue 4 (RB4), at 25 or 40 °C without redox mediators, reached 90%, 80% and 60%, respectively, using either pure Lacps1 or the crude extract. This is the first study of the decolorization of BPB and RB4 by a *P. sanguineus* laccase. The data suggested good potential for treatment of industrial dye-containing effluents.

## 1. Introduction

Laccases (benzenediol: oxygen oxidoreductases, EC 1.10.3.2) are multicopper oxidases broadly distributed in plants, insects, fungi and bacteria [1,2,3]. Most of them, blue colored, catalyze the direct oxidation of a wide variety of phenolic compounds [3,4]. Some yellow laccases, able to oxidize both phenolic and non-phenolic compounds, have also been described [5]. Besides the ability to oxidize a multitude of substrates, the laccases do not require hydrogen peroxide for the oxidation reaction; moreover, they use molecular oxygen as an electron acceptor, generating water. Therefore, they are considered ideal “green catalysts”, and are among the most promising oxidoreductases for application in different biotechnological processes in food, textile, pulp and paper, pharmaceutical and other industries [2,6].

The laccases are monomeric, dimeric or tetrameric proteins that contain four copper atoms per monomer, distributed in three redox sites named T1, T2 and T3 [5]. The redox potential (*E*°) of the T1 copper site is characteristic of each enzyme, ranging from 0.4 to 0.8 V *vs.* normal hydrogen electrode (NHE). According to it, the laccases are classified into three groups: low- (0.4–0.5 V), medium- (0.5–0.6 V) and high- (0.7–0.8 V) redox potential [7,8]. The *E*° is one of the most relevant characteristics of a laccase, since the redox potential difference between the T1 copper site and the substrate is one of the major factors that affect the oxidation rate. Furthermore, the *E*° of a laccase restrains the substrates that it may oxidize to those with lower *E*°. This limitation may be overcome by using redox mediators, which increase the oxidative capacity of the enzyme towards the oxidation of higher *E*° compounds; however, the industrial use of mediators is still hampered by their prohibitive cost and toxicity [4,7,8,9]. Therefore, high-redox potential laccases are more attractive for biotechnological purposes.

The increasing volume and chemical complexity of synthetic dye-rich industrial effluents, particularly from textile, tannery and pulp and paper plants, represent a serious environmental problem today [5,10,11]. Synthetic dyes often have low biodegradability, several are toxic, carcinogenic and mutagenic and it is not uncommon that their degradation products show similar properties. Furthermore, the absorption and reflection of the sunlight by dyes in surface waters interferes with bacterial and plant growth, disturbing the ecological balance [9,11,12]. As a result, there is a great interest in the development of efficient treatments for the removal of synthetic dyes from wastewaters.

Various physicochemical methods for dye removal are available, but they are costly, poorly efficient and not applicable for a variety of compounds. Microbiological processes are relatively inexpensive, in contrast, and environmentally friendly; however, their use depends on the identification of suitable microbial strains and the establishment of adequate conditions for dye degradation, often requiring previous physical and/or chemical steps. Furthermore, numerous synthetic dyes are resistant to microbiological attack. Thus, the use of oxidative enzymes is now considered the most promising alternative for the removal of dyes from industrial effluents [9,10,11,13,14]. The large-scale use of laccases for wastewater treatments is, however, severely limited by their high production costs. This has lately generated several studies on the optimization of the culture conditions for laccase production by different fungi in low-cost media [14,15,16].

The fungi from the phylum Basidiomycota are among the most efficient laccase producers known [1,3,4]. Among them, the white-rot fungus, *Pycnoporus sanguineus*, has attracted considerable attention in recent years due to its ability to produce high levels of high redox potential laccases as the main ligninolytic enzymes [7,17,18,19]. Furthermore, laccases from different *P. sanguineus* strains revealed good potential for application in industrial dyes bioremediation, detoxification and decolorization processes [7,17,19,20].

Several authors have studied the production of laccases by different strains of *P. sanguineus* under submerged fermentation (SmF) conditions [21,22,23]. In the last decade, however, solid state fermentation (SSF) processes were consolidated as an interesting alternative to SmF for the production of industrial enzymes, particularly from fungal origin [24,25,26,27]. The main advantages of SSF include higher yields and the production of enzymes that are not produced under SmF, and are attributed to the conditions in which the microorganisms are cultivated, similar to those of their natural environment [24,26]. Furthermore, SSF processes have several potential economic and environmental advantages: different agro industrial by-products and residues may be used as solid substrates, lowering the production costs and avoiding pollution issues related to waste disposal; the effluent generation and the demands on energy and sterile water are lower; the enzymes are obtained at higher concentrations, reducing downstream processing [24,26].

In spite of that, few studies have investigated the production of laccases by *P. sanguineus* under SSF conditions, employing the agro residues sago “hampas”, rubber wood sawdust and oil palm frond parenchyma tissue (OPFPt), abundant in Malaysia [28,29,30]. A recent study has also investigated laccase production using *Eucalyptus* *grandis* bark shavings, an abundant residue in Uruguay, but in this case the fungus was cultivated under semi-solid-state fermentation (semi-SSF) conditions, a variety of SSF with higher free liquid content in the culture medium [31].

In this study, we investigated the potential of different agro-industrial residues and by-products that are abundant in Brazil as carbon sources for the production of laccases by the newly isolated strain *P. sanguineus* RP15 under SSF conditions. The culture conditions for enzyme production were optimized using response surface methodology (RSM) and a major laccase (Lacps1) was purified and characterized in detail. The potential of the laccase-rich crude culture extract and the pure enzyme for the decolorization of the synthetic dyes remazol brilliant blue R (RBBR), reactive blue 4 (RB4) and bromophenol blue (BPB) was evaluated.

## 2. Results and Discussion

### 2.1. Preliminary Screening for the Best Carbon Source, Inducer and Supplementary Nitrogen and Carbon Sources for the Production of Laccases by P. sanguineus RP15

The best carbon source for laccase production by *P. sanguineus* RP15 was wheat bran, reaching 29.8 ± 1.9 U·g^−1^ (Table 1). Negligible enzyme levels were produced in peanut hulls and rice husks, and the organism was not able to grow on corn husks. Laccase activity was undetectable in the crude extracts obtained after *P. sanguineus* culture in all other carbon sources tested. Under SmF conditions, wheat bran was a poor carbon source for laccase production by *P. sanguineus* CS2 [21]. In contrast, the lignocellulosic agro-residues sago “hampas”, rubber wood sawdust and oil palm frond parenchyma were good carbon sources for the production of laccases by *P. sanguineus* CY788 under SSF conditions [28,29,30], while *Eucalyptus* bark shavings were revealed to be a good solid substrate for enzymatic production by a Uruguayan strain of *P. sanguineus* under semi-SSF conditions [31]. Similarly, a variety of lignocellulosic waste materials or by-products derived from food and agricultural processing industries have been successfully used as solid substrates for the production of laccases by other white-rot basidiomycetes under SSF conditions [32,33,34,35,36]. As observed for *P. sanguineus* RP15, wheat bran has been successfully employed as the main or sole carbon source for the production of laccases by *Fomes* *sclerodermeus* [37], *Ganoderma* sp. [38] and *Fomes* *fomentarius* [39] under SSF conditions, possibly due to the high content of cellulose, hemicelluloses and lignin as well as growth factors, vitamins and proteins [40,41].

The addition of veratryl alcohol (0–10.0 mmol^−1^) to wheat bran slightly inhibited the laccase production by *P. sanguineus* RP15 with respect to the control (wheat bran and water only). The inhibition was concentration-dependent and a decrease of about 23% occurred at 10 mmol·L^−1^ veratryl alcohol. In contrast, the addition of CuSO_4_ (0–100 mmol·L^−1^) resulted in enhanced laccase production, reaching 37.2 ± 2.1 U·g^−1^ (17% above the control) at 50 mmol·L^−1^. At higher concentrations, however, a steady decrease of the enzymatic production occurred and 66% lower levels, as compared to the control, were obtained at 100 mmol·L^−1^ CuSO_4_. The production of fungal laccases is often influenced by a series of inducers, which apparently regulate the enzyme synthesis at transcriptional level. Aromatic and phenolic compounds, especially those structurally related to lignin, and metallic ions are known inducers of laccase expression. However, the effect of each compound/ion and the optimum concentration for laccase production are highly variable among different fungal strains [1,4,36,42]. Veratryl alcohol and Cu^2+^ are among the most common inducers of fungal laccases, and their effect on the enzymatic production by various strains under SmF conditions is well characterized [4,42]. Few studies, however, addressed this point under SSF conditions. Contrasting to our results, the production of laccases by *Pycnoporus cinnabarinus* ss3 [43] and *Trametes versicolor* [16] cultured in sugarcane bagasse and horticultural waste, respectively, was increased about nine- and three-fold, respectively, by supplementation with veratryl alcohol. Furthermore, laccase production levels by *Fomes* *fomentarius* WRF-1 [39] and *Ganoderma* sp. [38] cultured in wheat bran were highly enhanced in response to the addition of CuSO_4_. Slight inhibition was observed, however, when cultures of *Fomes* *sclerodermeus* in wheat bran were supplemented with micromolar concentrations of CuSO_4_ [37].

The supplementation of wheat bran with 1% (*w*/*w*) milled corncob resulted in an increase of about 18% of the laccase production by *P.* *sanguineus* RP15 (Table 1). With the exception of soybean meal and corn husks, which slightly reduced the production, all other supplementary carbon sources tested were practically without effect. Up to date, the use of corncob as the main or sole carbon source for laccase production by *Pycnoporus sanguineus* has not been investigated. Furthermore, few authors have studied the use of this cheap agro-residue for laccase production by other basidiomycetes, although it should provide conditions for enzyme induction due to its lignocellulosic nature [44]. Low production levels in media containing corncob as the carbon source have been described for *Pleurotus florida* EM 1303 [44], *Lentinula* *edodes* CCB-42 [45] and *Trametes versicolor* IBL-04 [46].

The best supplementary nitrogen sources for the production of laccases by *P. sanguineus* RP15 decreased in the following order: NH_4_Cl > peptone > malt extract > casein > urea > yeast extract > asparagine > KNO_3_ > NH_4_NO_3_ > (NH_4_)_2_SO_4_ > NaNO_3_. The production was increased about 37% by 1.0% (*w*/*w*) peptone and 68% by 0.8% (*w*/*w*) NH_4_Cl, while it was inhibited about 50% by 0.8% (*w*/*w*) NaNO_3_ (Table 1). Thus, the quality of the nitrogen source has strongly influenced the laccase production in wheat bran, a complex substrate that presents high nitrogen levels [47]. Furthermore, when NH_4_Cl was added to wheat bran at variable concentrations (0.0%–5% *w*/*w*), the highest laccase levels were produced by *P. sanguineus* RP15 at 0.8%–1.0% (*w*/*w*). Contrasting to our results, the highest laccase production by *Phlebia* *floridensis* under SSF in wheat straw was obtained with a mixture of 9% (*w*/*w*) NH_4_Cl and 5% (*w*/*w*) malt extract as nitrogen sources [48].

### 2.2. Optimization of Laccase Production by P. sanguineus RP 15 Using RSM

After preliminary studies to determine the experimental ranges for each independent variable (culture time, initial moisture of the medium, temperature and milled corncob concentration) the culture conditions for the production of laccase were optimized using a 2^4^ full-factorial Central Composite Rotational Design (CCRD) (Table 2). The maximum and minimum laccase levels were, respectively, 137.2 ± 14.2 U·g^−1^ (run 26) and 29.4 ± 2.4 U·g^−1^ (run 21).

From the results of the CCRD, the variables that exerted significant effects on laccase production at 95% confidence level (*p* < 0.05) were identified. The linear terms of temperature and milled corncob concentration, and the quadratic terms of culture time, temperature and initial moisture of the medium, exerted statistically significant effects on the enzymatic production. The interaction between the initial moisture of the medium and the temperature also had a significant effect, but not the other interactions between the independent variables tested. An ANOVA analysis was performed considering only the significant effects and the regression coefficients obtained are presented in Table 3. The quadratic effects of the initial moisture of the medium and the temperature were the most important factors that affected the production of laccases.

The second-order model that describes the production of laccases by *P. sanguineus* as a function of the independent variables tested was expressed by the equation:

$$\text{Lac }\left(U\cdot g^{-1}\right)=\left[124.33\;+\;9.72\;z+8.10\;w-12.54\;x^{2\;}-23.42\;y^{2\;}-20.04\;z^{2\;}+\;11.25\;zy^{\;}\right]$$

Based on the *F* test, the model was predictive of the production of laccases as a function of culture time, culture temperature, medium initial moisture and milled corncob concentration, since the calculated *F* value was higher than the listed one. The *R*^2^ coefficient of 0.80 confirmed the goodness of the model and indicated that it could explain 80% of the response variability.

The statistical analysis showed that in a medium composed of wheat bran as the carbon source supplemented with 0.8% (*w*/*w*) NH_4_Cl and 50 mmol·L^−1^ CuSO_4_, the maximum laccase production occurred after 8.19 culture days at 26.2 °C, with initial moisture of 4.10 mL·g^−1^ and addition of 17.98% (*w*/*w*) milled corncob as the supplementary carbon source. The maximum predicted laccase production was 140.78 U·g^−1^, about 4.7-fold higher than that obtained in wheat bran and water (29.8 ± 1.9 U·g^−1^).

**Figure 1:**
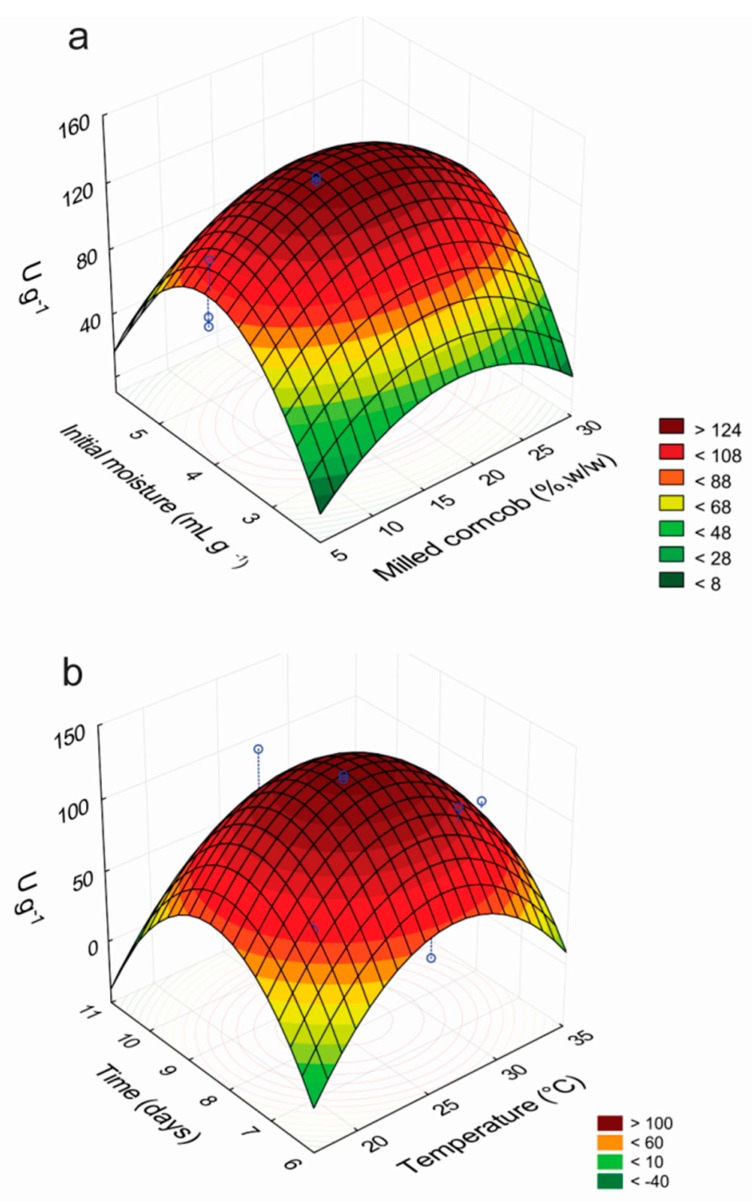
Response surface plots showing the interactive effects of initial moisture and milled corncob concentration (**a**), and temperature and culture time (**b**), on the production of laccases by *P. sanguineus* RP15.

For the experimental validation of the optimized culture conditions, the fungus was grown for eight days at 26 °C in wheat bran supplemented with 0.8% (*w*/*w*) NH_4_Cl, 50 mmol·L^−1^ CuSO_4_ and 18% (*w*/*w*) milled corncob, with initial moisture of 4.1 mL·g^−1^, and the mean value determined for the laccase activity was 138.6 ± 13.2 U·g^−1^, in very good correlation with the value predicted by the model, confirming its validity. The response surfaces for the factors that affected the production of laccases by *P. sanguineus* RP15 are presented in Figure 1. The analysis of the curves revealed that the best conditions for the enzymatic production are in the ranges of 7.5–9.0 culture days, temperature of 25.5–27.5 °C, medium initial moisture of 3.5–4.5 mL·g^−1^ and milled corncob concentration of 15%–25% (*w*/*w*).

The relatively short culture period, the low-cost salts employed as the supplementary nitrogen source (NH_4_Cl) and inducer (CuSO_4_), and the use of about 20% milled corncob as the supplementary carbon source constitute definite advantages of the culture medium employed for laccase production by *P. sanguineus* RP15. In particular, corncobs constitute one of the most abundant and cheap residues of the agricultural industry around the world [49] and the carbon source employed in a certain enzyme production process constitutes about 40%–60% of the total costs [15].

Few authors have investigated the production of laccases by *P. sanguineus* strains under conditions of SSF [28,29,30] and semi-SSF [31]. The main carbon sources used in these studies were lignocellulosic wastes and/or agro-industrial by-products, but only Annuar *et al.* [30] and Gioia *et al.* [31] performed the statistical optimization of the enzymatic production. Gioia *et al.* [31] employed ABTS to estimate the laccase activity, and under optimal conditions their *P sanguineus* strain produced 106 U·g^−1^ solid substrate, about 25% less than *P. sanguineus* RP15. Furthermore, in spite of the low cost of the culture medium employed (bark shavings with the addition of salts, thiamine and yeast extract), the culture period for maximal enzyme production (14 days) was much higher than that determined for *P. sanguineus* RP15, increasing the production costs.

In contrast to *P.* *sanguineus*, the production of laccases by other white-rot fungi species under SSF conditions has been investigated by several authors. From three- to twenty-fold lower production levels, as compared to that obtained for *P. sanguineus* RP15, were reported for different species of the genus *Pleurotus* [33,34,50,51] and for *T. versicolor* [16,51]. In great contrast, a 72-fold higher production, as compared to that obtained in this study, was described for an isolate of *Ganoderma* sp. [38]. Levels about two- and six-fold higher were also found for strains of *F. sclerodermeus* [37] and *Phanerochaete chrysosporium* [15], respectively. However, these higher productivities were obtained in more expensive culture media, as compared to that of *P. sanguineus* RP15, and/or the culture times were much higher (from two- to three-fold), contributing to an increase in the production costs.

### 2.3. Lacps1 Purification and Molecular Properties

Lacps1 was purified by a simple two-step method from the crude culture extract from *P. sanguineus* RP15 cultivated under optimized conditions for laccase production (Table 4). The enzyme was purified about 24.5-fold with an overall yield of 30%, reaching a specific activity of 411.0 U·mg^−1^. When maintained in water at 4 °C, the activity of Lacps1 remained constant for at least 60 days.

**Figure 2:**
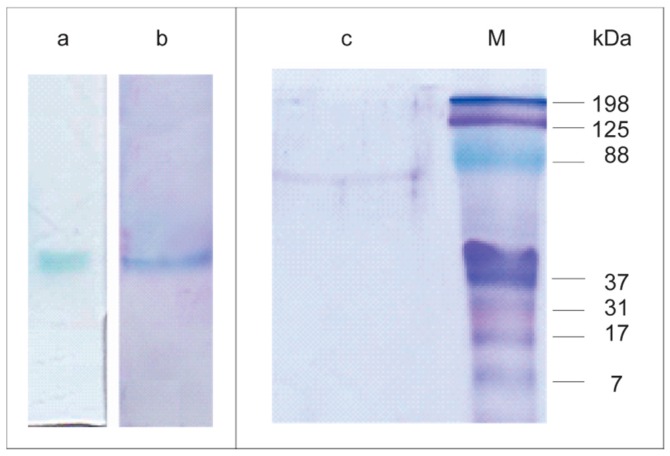
Analysis of the purified laccase by PAGE (lanes **a** and **b**) and SDS-PAGE (lane **c**). Lane **a**, laccase activity after PAGE, using 1 mmol·L^−1^ 2,2’-azino-*bis*(3-ethylbenzothiazoline-6-sulfonate) (ABTS) as substrate; lanes **b** and **c**, protein staining. Total protein loading was 25 µg per lane. M, molecular mass markers.

Non-denaturing polyacrylamide gel electrophoresis (PAGE) of the purified Lacps1 revealed a single laccase activity band (Figure 2a), coincident with a single band after Coomassie Blue staining (Figure 2b), confirming the homogeneity of the preparation. A single protein band corresponding to 65 kDa was also revealed after sodium dodecyl sulfate-PAGE (SDS-PAGE) analysis (Figure 2c). The apparent molecular mass of Lacps1 estimated by gel filtration was 67 kDa, in good agreement with that estimated by SDS-PAGE, suggesting that the native enzyme was a monomer. Similar apparent molecular masses were reported for several laccases from other *P. sanguineus* strains [7,17,18,19,52,53,54,55,56] and other *Pycnoporus* species [7,20].

Most extracellular fungal laccases are glycoproteins, and their carbohydrate content usually ranges from 10% to 30%. Laccases with higher saccharide content, however, have been described [2,4,7]. Lacps1 showed a carbohydrate content of about 24%, close to those determined for two laccases from *P. sanguineus* CS43 [19], but about three-fold higher than those estimated for the laccases from *P. sanguineus* BRFM66 and *P. sanguineus* BRFM902 [7]. The enzyme-bound carbohydrate moieties of fungal laccases greatly affect their properties, such as proteolytic susceptibility, copper retention ability, kinetic parameters, optimum pH and thermal stability [19,56].

Lacps1 was characterized by mass spectrometry (MS) after trypsinolysis. Three tryptic peptides were detected, and their respective precursor ion masses and amino acid sequences were: *m*/*z* 1,242.682 (SPGTTAADLAVIK), *m*/*z* 1718.75 (SAGSSEYNYDNPIFR) and *m*/*z* 1992.69 (ANPSFGNTGFAGGINSAILR). The results of the mass spectrometry analysis and database search demonstrated that the identified peptides shared 100% sequence identity to peptides of two laccases from *Pycnoporus coccineus* M85-2 (UNIPROT: Q96TR6 and Q96VA5) [57]. The amino acid sequences of the tryptic peptides covered about 9.3% of the amino acid sequences of both *P. coccineus* laccases.

### 2.4. Effects of pH and Temperature on Laccase Activity

The effects of pH and temperature on the enzymatic activity of Lacps1 were evaluated using a CCRD and RSM analysis. The experimental conditions and the results of the experimental design are summarized in Table 5.

The statistical analysis of the data revealed extremely low *p*-values for the linear and quadratic effects, and also for the interaction between the pH and temperature, indicating their significant effects on the enzymatic activity (Table 6).

The quadratic model that describes the laccase activity of Lacps1 as a function of the reaction pH and temperature was expressed by the equation:

$$U\cdot {\text{mg}}^{-1}=[396.25-47.99\text{ pH}-20.24\text{ T}-135.57{\text{ pH}}^{2}-89.72{\text{ T}}^{2}\;+18.23\text{ pH}.T]$$

The ANOVA for the model (Table 6) revealed that, based on the *F* test, it was predictive of the laccase activity, since the calculated *F* value was much higher than the listed *F* value. The *R*^2^ coefficient (0.99) confirmed the goodness of the model.

**Figure 3:**
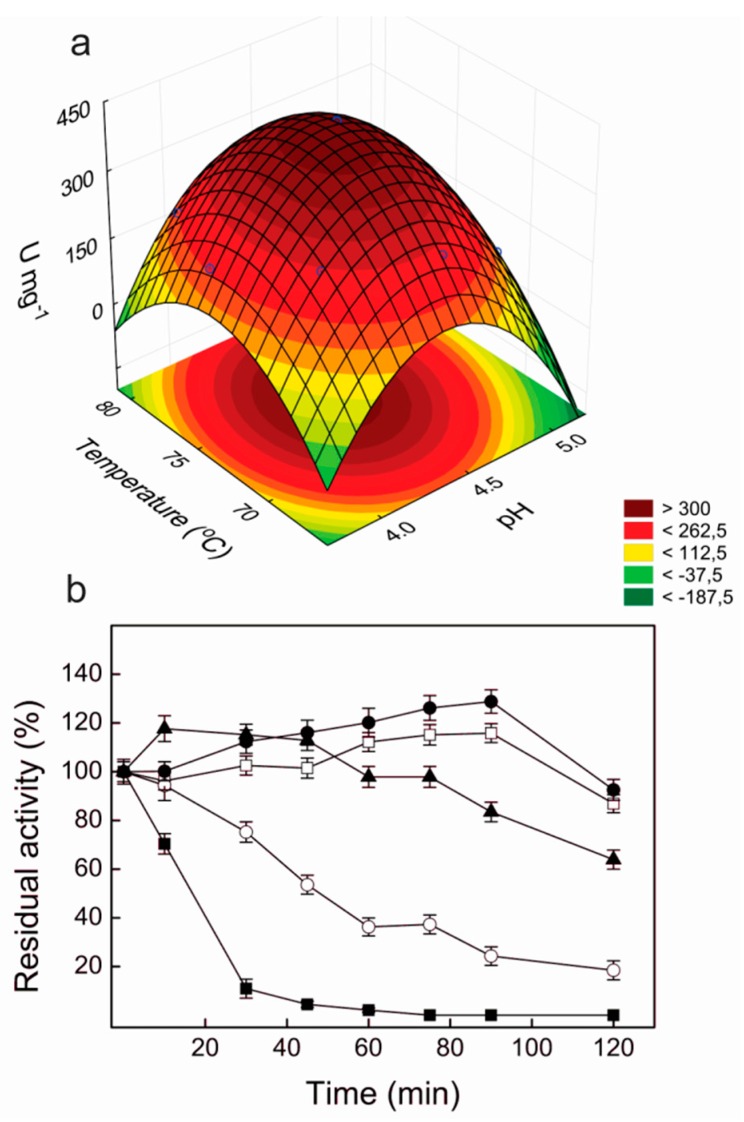
Response surface curve for the effects of pH and temperature on laccase activity (**a**), and thermal stability of the purified Lacps1 (**b**). In thermal stability studies (**b**), samples of the purified laccase in water were incubated at 55 °C (□), 60 °C (●), 65 °C (▲), 70 °C (O) or 75 °C (■), and the residual activities were determined at 65 °C in McIlvaine buffer, pH 4.5, containing 1.0 mmol·L^−1^ ABTS. One hundred percent specific activity corresponded to 401.4 ± 17.9 U·mg^−1^. The values presented correspond to means ± SD from triplicate experiments (*n =* 3) performed with three separate preparations of pure Lacps1.

According to the statistical analysis, the optimum pH and temperature for the laccase activity were 4.4 and 74.4 °C, respectively, with a maximum predicted value of 402.4 U·mg^−1^. A very close value was determined in the experimental validation (397.1 ± 20.8 U·mg^−1^), confirming the validity of the model. The response surface curve for the effects of pH and temperature on the laccase activity of Lacps1 is illustrated in Figure 3a; useful pH and temperature working ranges corresponded to 4.0–4.75 and 70–79 °C, respectively.

Among the purified laccases from *P. sanguineus* described to date, most presented optimum pH for the oxidation of ABTS in the range of 2.0–3.0 [17,19,53,54,58], and similar values were determined for two laccases from *Pycnoporus* sp. SYBC [20]. However, the enzymes from *P. sanguineus* BRFM66 and *P. sanguineus* BRFM902 [7] exhibited maximum activity at pH 4.0–5.0, similarly as observed for Lacps1. In general, the laccases from other white-rot basidiomycetes exhibit pH optima for the oxidation of ABTS in the acid range (2.2–5.0) [18,59,60,61,62,63,64,65,66].

The optimum temperature for ABTS oxidation by Lacps1 was somewhat higher than those reported for the laccases from other *P. sanguineus* strains, that varied from 50 to 70 °C [7,17,19,53,54], which is an attractive feature for application in some biotechnological processes. Optimal temperatures in the range 40–70 °C were found for laccases from other white-rot fungi [59,60,61,62,63,64,65,66].

### 2.5. Thermal and pH Stabilities of Lacps1

Lacps1 was fully stable over the pH range from 3.0 to 8.0, with residual activities of about 57% and 64% at pH 2.0 and 10.0, respectively. Furthermore, the pure enzyme maintained full activity for 120 min at 55 and 60 °C, and retained about 64% of the initial activity after 120 min at 65 °C. Half-lives of 45 min and 16 min were determined at 70 and 75 °C, respectively (Figure 3b).

In contrast to the wide pH stability of Lacps1, two laccases from *P. sanguineus* CS43 were stable only in the range from pH 6.0 to 8.0 [19], while a laccase from a Chinese isolate of *P. sanguineus* showed good stability only in the acidic pH range (2.0–5.0) [17]. Likewise, the laccases from *Pleurotus* sp. [60] and *Ganoderma* *lucidum* [61] were stable in a restrict pH range (3.0–6.0). Similarly to Lacps1, various laccases from different *P. sanguineus* strains show good thermal stability [7,53,54], although some less stable laccases from this fungus have been described [17,19,55]. The good thermal stability and wide pH tolerance of Lacps1 are valuable properties for biotechnological applications, since at certain stages of some industrial processes the enzymes may be, even temporarily, exposed to harsh conditions, such as elevated temperatures and/or pH conditions far from those required for their maximal activity and stability.

### 2.6. Kinetic Properties and the Effects of Metals and Inhibitors on the Laccase Activity

The stimulation of the activity of Lacps1 by ABTS followed Michaelian kinetics, with a maximum velocity (*V*_M_) of 413.4 ± 21.2 U·mg^−1^. The Michaelis-Menten constant (*K*_M_) value corresponded to 147.0 ± 7.4 μmol·L^−1^ and the turnover number (*k*_cat_) value was 462.4 ± 2.4 s^−1^, resulting in a catalytic efficiency (*k*_cat_/*K*_M_) of about 3140.1 ± 149.6 L·mmol^−1^·s^−1^. Markedly different parameters were determined for a laccase produced by the same strain in wheat bran and water only, which oxidized ABTS with a *K*_M_ about 3.5-fold higher and a 10-fold lower *k*_cat_ value, at 25 °C, resulting in a catalytic efficiency around 88 L·mmol^−1^·s^−1^ [67]. Lower *k*_cat_ values (1.15 to 236.9 s^−1^), as compared to that determined for Lacps1, were also found for the laccases from other *P. sanguineus* strains [7,17,53,54,55,56] and other white-rot fungi [61,62,63,68]. Remarkable exceptions are, however, two laccases from *P. sanguineus* CS43 [19], one from *Pycnoporus* sp. SYBCL1 [20] and two from different *Trametes* species [64,65], with similar or higher *k*_cat_ values. The *K*_M_ values for the oxidation of ABTS by the laccases from different *P. sanguineus* strains are highly variable (12 to 238 μmol·L^−1^) [7,17,19,53,54,55,56]. The value estimated for Lacps1 lies in the upper half of this range, resulting in a relatively low catalytic efficiency for ABTS oxidation, as compared to some other enzymes.

The activity of Lacps1 was practically unaffected by the presence of Ca^2+^, Sr^2+^, Co^2+^, Zn^2+^, Ni^2^, Mg^2+^ and Na^+^ at 1 and 5 mmol·L^−1^ concentrations. In contrast, the enzyme was highly sensitive to Hg^2+^, Ag^+^ and Fe^2+^, with residual activities of 17.0%, 31.6% and 22.4%, respectively, at 1 mmol·L^−1^ concentration and total depletion at 5 mmol·L^−1^ concentration. In addition, Pb^2+^ completely abolished the activity, at both concentrations tested. Similar inhibition patterns by divalent metal ions were observed for the laccases from *Pycnoporus* sp. SYBCL1 [20] and other white-rot fungi [60,69].

Differently, Cu^2+^ stimulated about 12.7% and 31.5% the activity of Lacps1, at concentrations of 1 and 5 mmol·L^−1^, respectively. Similarly to our results, stimulation by Cu^2+^ has been described for two laccases from *Pycnoporus* sp. SYBC-L1 [20], as well as for the laccases from some other white-rot fungi [59,66,70]. The stimulation by Cu^2+^ has been attributed to the filling of the T2 copper sites on the enzyme molecules [66]. However, some Cu^2+^-inhibited laccases have also been described [69]. 

Lacps1 was fairly resistant to SDS: residual activities of 78% and 17.3% were estimated in the presence of the anionic detergent at 1 and 5 mmol·L^−1^ concentrations, respectively. In contrast, most laccases from white-rot fungi were strongly inhibited by SDS [20,60]. Ethylenediaminetetraacetic acid (EDTA) and sodium azide completely depleted the activity of Lacps1 at 1 mmol·L^−1^ concentration, and a residual activity of about 10% was observed in the presence of 1 mmol·L^−1^ dithiothreitol (DTT); the reducing agent completely inhibited the activity at a 5 mmol·L^−1^ concentration. Inhibition by thiol compounds, azide and EDTA has been extensively described for white-rot fungi laccases [1,20,60,66,69,71], including two enzymes from *P. sanguineus* strains [17,52]. However, some EDTA- and DTT-insensitive laccases have been identified [20,60,69,71]. Azide is considered a true laccase inhibitor and seems to bind to the T2 and T3 copper sites, interfering with the internal electron transfer and decreasing the catalytic activity [69,71]. The inhibition of laccases by DTT is less understood. According to some authors, it results from the coordination of the thiol reagent to the copper atoms at the active site [69]. However, there is also some evidence that the inhibition could be a secondary effect, derived from a reduction of the reaction product, ABTS^+^, by DTT [71].

### 2.7. UV–Vis Absorption Spectrum of Lacps1

**Figure 4:**
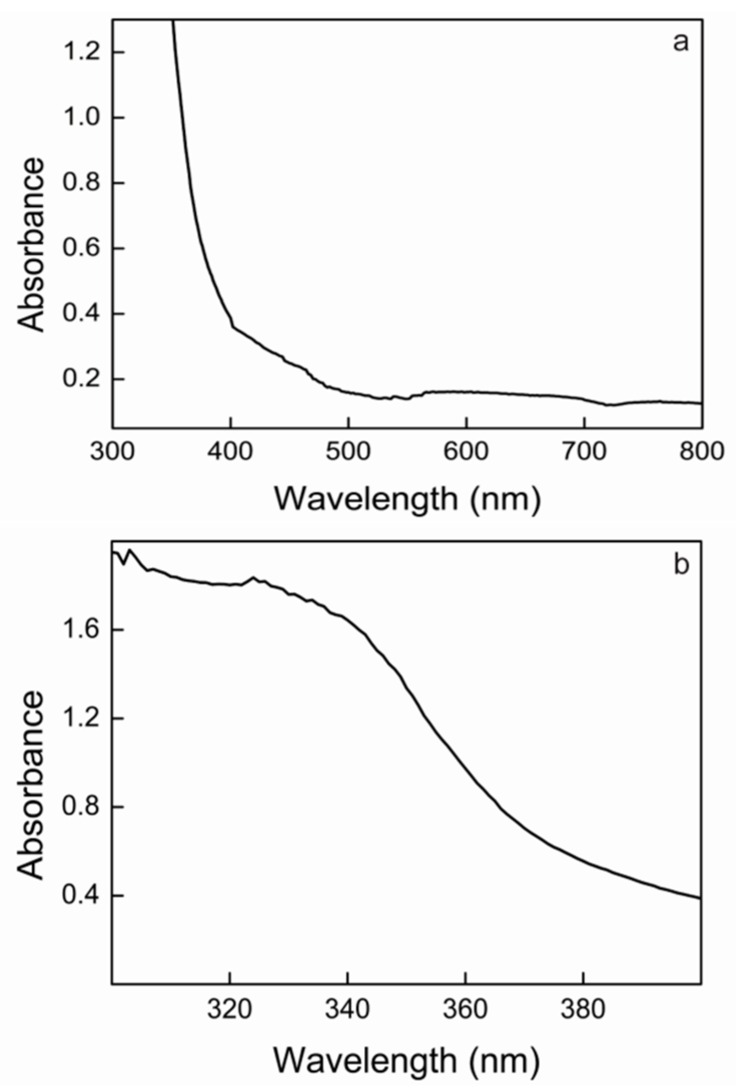
UV–Vis spectrum of pure Lacps1 (**a**), and detail of the range from 300–400 nm (**b**). The experiment was repeated three-fold with three separate preparations of pure Lacps1; representative spectra are presented.

Concentrated solutions of Lacps1 exhibited the typical color of the blue laccases. In agreement, the absorption spectrum exhibited a peak around 600 nm, indicating the presence of a T1, or blue copper, atom (Figure 4a). The shoulder seen in the spectrum at around 300 nm suggested the presence of a T3 copper site in the enzyme molecule (Figure 4b). Highly similar absorption spectra have been obtained for other blue laccases from white-rot fungi [19,69].

### 2.8. Determination of the Redox Potential of the T1 Copper Site of Lacps1

At the concentration of 0.2 mmol·L^−1^, the mediator Fe(dipyridyl)_2_Cl_2_ has not significantly interfered with the “blue” band of the copper II ion at the T1 site of Lacps1. As the concentration of Fe(dipyridyl)_2_Cl_3_ was increased, the reduction of the copper II was evidenced by the progressive disappearance of the “blue” absorbance band over 550–800 nm, and from the resulting Nernst plot a one-electron reduction was observed with an *E*° of 0.747 V *vs.* NHE for the T1 copper site. Thus, Lacps1 belongs to the group of high-redox potential laccases, an attractive feature for different biotechnological applications. Comparable *E*° values (0.72–0.75 V *vs.* NHE) have been determined for laccases from other *P. sanguineus* strains [7] and also from *P. coccineus* BRFM 938 [7] and some *Trametes* strains [72].

### 2.9. Dye Decolorization by Lacps1

**Figure 5:**
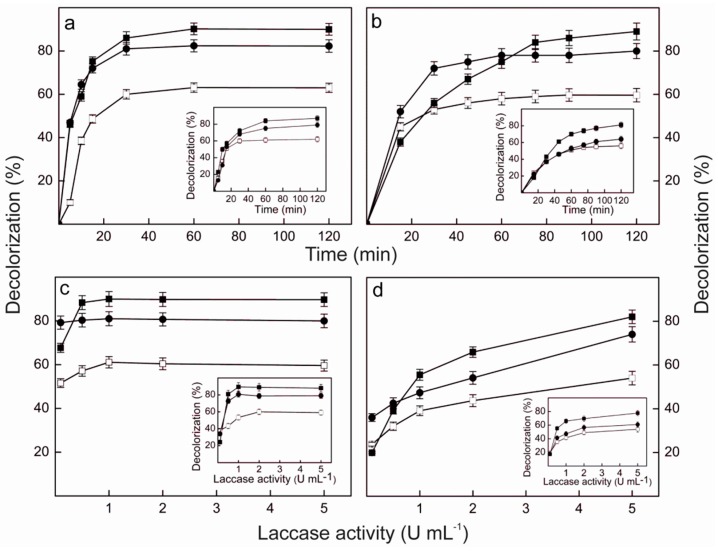
Effects of the reaction time and the laccase activity load on the decolorization of BPB, RB4 and RBBR by the pure Lacps1 (**a**,**c**), or the crude culture extract from *P. sanguineus* RP15 (**b**,**d**), at 40 °C. Dyes final concentrations were 100 mg·L^−1^ (RBBR and RB4) or 25 mg·L^−1^ (BPB), and the reactions were carried out in McIlvaine buffer, pH 4.5. (**a**,**b**). The enzymatic load was 5 U·mL^−1^ (**c**,**d**); the reaction time was 120 min. Insets: Effects of the reaction time (**a**,**b**), and the laccase activity load (**c**,**d**), on dye decolorization by the crude extract (**b**,**d**), or the pure Lacps1 (**a**,**c**), at 25 °C. Data are presented as means ± SD from triplicate experiments (*n* = 3) performed with three separate preparations of crude extract or pure Lacps1 (error bars are not evident, as they lie within the area of the symbol). Symbols: BPB (■), RBBR (●) and RB4 (□).

The ability of the pure Lacps1 and the laccase-rich crude extract from *P. sanguineus* RP15 to degrade RBBR, RB4 and BPB was analyzed at 25 and 40 °C (Figure 5), temperatures in which preliminary experiments revealed that the enzyme showed good levels of activity on the dyes and excellent thermal stability. The temperature exerted little effect on the maximum levels of decolorization, when the pure enzyme was employed (Figure 5a,c). After 120 min reaction with an enzyme load of 5 U·mL^−1^, the percentages of decolorization reached about 90%, 80% and 60% for BPB, RBBR and RB4, respectively, either at 25 °C (insets to Figure 5a,c) or 40 °C. However, at 40 °C the maximum decolorization of BPB and RBBR was attained after 30 min (Figure 5a), while 60 min were required at 25 °C (inset to Figure 5a). In contrast, RB4 was maximally decolorized after 30 min at both temperatures. The reaction temperature also affected the enzyme load needed to attain the maximum level of decolorization after 120 min (Figure 5c). While at 40 °C it corresponded to 0.5 U·mL^−1^ for BPB, 0.1 U·mL^−1^ for RBBR and 1.0 U·mL^−1^ for RB4, at 25 °C about 1.0 U·mL^−1^ was required for BPB and RBBR, and 2.0 U·mL^−1^ for RB4 (inset to Figure 5c).

BPB is a triphenylmethane dye extensively used in textile, paper, leather, food and cosmetic industries [73]. Among the different dye groups, the triphenylmethane dyes are particularly resistant to enzymatic treatments and long reaction times are usually required for, sometimes, even a partial decolorization. To the best of our knowledge, this is the first study on the decolorization of BPB by the action of a laccase from *P. sanguineus*, either in pure or crude form. In great contrast to our results, a maximum decolorization level of BPB around 20% was obtained using 8 U·mL^−1^ of a pure commercial laccase from *T. versicolor* after three hours at 35 °C [74], while maximum levels of about 90% were obtained with 6.5 U·mL^−1^ of a pure laccase from *Trametes* *trogii* 463 at 30 °C, but after 24 h [75]. However, similarly as observed for Lacps1, a decolorization level of about 90% was achieved after treatment of BPB with 5 U·mL^−1^ of a pure laccase from *Lentinula edodes* for 90 min at 30 °C [70].

RB4 and RBBR are typical reactive anthraquinonic dyes, widely used in the textile industry. The dyes of this class are especially resistant to degradation due to their fused aromatic rings, and most are toxic, carcinogenic and mutagenic. Furthermore, under usual dyeing conditions, up to 50% of the reactive dye initially present is discharged, generating highly colored effluents [12,20,76,77]. In spite of its unquestionable interest, the degradation of RB4 by other crude or pure laccases from *P. sanguineus* has not been investigated to date. However, similar to our results, maximum levels of RB4 decolorization of about 69% were attained after 30 min at 30 °C using a laccase-rich crude extract from *T.* *trogii* SYBC-LZ at an enzyme load of 1.1 U·mL^−1^ [78].

In great contrast, the degradation of RBBR by purified laccases has been under intense investigation in the last years. Considering the enzymes produced by different species or strains of *Pycnoporus*, maximum levels of decolorization comparable to those determined for Lacps1 were obtained with a laccase from a Chinese isolate of *P. sanguineus*, under similar conditions of enzyme load, reaction time and temperature [17]. In contrast, a maximum decolorization of 20% was reached under the same conditions when a laccase from *Pycnoporus* sp. SYBC-L1 was employed [20], and 52 h were necessary to attain around 90% decolorization using each of three purified laccases from *P. coccineus*, at an enzyme load of 0.1 U·mL^−1^ [7]. Among the enzymes from other white-rot fungi, similar maximum percentages of decolorization, as compared to Lacps1, were obtained with the purified laccases from *Trametes* sp. SQ01 [79] and *Cerrena* sp. HYB07 [80], under comparable reaction conditions. Maximum decolorization levels of 80%–90% have also been obtained with the laccases from *Lentinus* sp. [13], *T. versicolor* [74] and *Armillaria* sp. F022 [81], but much higher enzyme loads or reaction times were required. In contrast, a maximum decolorization of about 15% was obtained with a laccase from *Ganoderma* sp. rckk-02, even after 12 h with an enzyme load of 10 U·mL^−1^ [82]. Taken together, these comparisons reveal that Lacps1 is among the most efficient white-rot fungi laccases known to date for the decolorization of BPB, RBBR and RB4.

The patterns of decolorization of BPB, RBBR and RB4 obtained using the laccase-rich crude extract from *P. sanguineus* RP15 (Figure 5b,d) were similar to those obtained with the pure Lacps1. The reaction temperature exerted little effect on the maximum levels of decolorization, which were close to those obtained with the pure enzyme: after 120 min with 5 U·mL^−1^ of laccase activity the percentages of decolorization reached 80%–90% for BPB, 70%–80% for RBBR and 50%–60% for RB4, both at 25 °C (insets to Figure 5b,d) and 40 °C. At 40 °C, however, the maximum decolorization of RBBR and RB4 was achieved after 45 min (Figure 5b), while about 90 min and 75 min were needed at 25 °C, respectively (inset to Figure 5b). Furthermore, the percentage of decolorization of BPB did not reach a well-defined maximum after 120 min, at both temperatures tested (Figure 5b and inset to Figure 5b). Similarly, the percentages of decolorization of the three dyes have not reached maxima at 5 U·mL^−1^ (Figure 5d), suggesting that higher decolorization efficiencies could be achieved using higher laccase loads, both at 25 °C (inset to Figure 5d) and 40 °C. Similar to our results, maximal decolorization of RBBR and BPB (around 80%) occurred after 30 min at 30 °C using 6.5 U·mL^−1^ of a purified laccase from *T.* *trogii* BAFC 463 while a three-fold higher enzyme load was required to obtain a similar level of decolorization using the crude culture extract [75].

A comparison of the results obtained with the pure Lacps1 and the crude extract strongly suggested that the decolorization of BPB, RBBR and RB4 by the crude extract could be mostly attributed to the action of Lacps1, although other oxidases and natural redox mediators could have been present and contributed to the degradation, as proposed by other authors [75,83]. Furthermore, the excellent potential of the *P. sanguineus* RP15 crude extract for application in dye decolorization processes was undoubtedly demonstrated. Although somewhat higher enzyme loads and reaction times may be needed to attain the same decolorization efficiencies obtained with the pure enzyme, the use of crude laccase preparations at relatively low temperatures (25–40 °C) is much more economical for practical applications, minimizing energy consumption and avoiding expensive and time-consuming steps of enzyme purification [75,83]. In spite of all these considerations, however, the success of a practical application of either the pure Lacps1 or the crude culture extract from *P. sanguineus* RP15 for the treatment of industrial effluents containing BPB, RBBR and/or RB4 depends on the results of a toxicity test of the degradation products of these dyes. In fact, the identification of the metabolites produced during the decolorization and/or biodegradation of a certain dye, as well as the evaluation of their toxicity, are essential steps for an eventual application of a laccase, assuring the safety of the treated effluents [11,36,84,85].

## 3. Materials and Methods

### 3.1. Organism and Strain Maintenance

The *Pycnoporus* strain was isolated from basidiocarps developing on dead fallen logs at the Campus of the University of São Paulo, at Ribeirão Preto (São Paulo, Brazil), and classified as *Pycnoporus sanguineus* according to morphologic and microscopic features as described by Ryvarden [86] and Teixeira [87,88]. The isolated strain (*P. sanguineus* RP15) was maintained on potato dextrose agarPDA medium, at 25 °C, and subcultured periodically.

### 3.2. Preliminary Screening for the Best Carbon Source, Inducer and Supplementary Nitrogen and Carbon Sources for the Production of Laccases by P. sanguineus RP15 under SSF Conditions

Mycelial sections (0.25 cm^2^) taken from 8-day-old cultures of *P. sanguineus* in PDA medium were inoculated into a sterile solid state medium (5 g of a dry agro-industrial by-product or residue as carbon source, 10 mL deionized water) in 250 mL Erlenmeyer flasks. The flasks were incubated for 192 h at 25 °C and 70% humidity, monitored by a thermo hygrometer MT-240 (Minipa, São Paulo, Brazil). After the determination of the best carbon source, the effect of supplementation with various nitrogen and carbon sources on the production of laccases was examined, under the same culture conditions. The effects of the addition of the presumed laccase inducers CuSO_4_ (0.0–100 mmol·L^−1^) and veratryl alcohol (0.0–10 mmol·L^−1^) to the best carbon source were also investigated, under equal conditions. All the experiments were conducted in triplicate (*n =* 3), and the enzymatic assays were carried out in duplicate.

### 3.3. Response Surface Methodology for the Optimization of the Production of Laccases

After choosing the best carbon source, inducer, and supplementary nitrogen and carbon sources, the culture conditions for the production of laccases by *P. sanguineus* RP15 were optimized using a CCRD and RSM. The levels of the independent variables (culture time, medium initial moisture, temperature and milled corncob concentration) were defined according to a 2^4^ full-factorial central composite design (star configuration) with 8 axial and 3 central points (triplicate in the central point) which resulted in 27 experiments. The experimental ranges of each independent variable were previously determined using the OFAT (one factor at a time) methodology, and corresponded to 6–10 culture days, 2.0–6.0 mL deionized H_2_O per gram dry carbon source, 15–35 °C and 5.0%–25.0% (*w*/*w*) milled corncob. Sun-dried corncobs were milled at 30 mesh (0.595 mm) using a knife mill SL31 (Solab, Piracicaba, Brazil). The experiment was repeated three-fold (*n =* 3) and each enzymatic assay was carried out in duplicate. The average of the laccase production (U·g^−1^) was used as the response value. Three cultures of *P. sanguineus* under optimized conditions for the production of laccases were performed to validate the optimization.

### 3.4. Enzyme Extraction

After growth, the *P. sanguineus* culture medium was suspended in 30 mL cold water and filtered through a nylon sieve after gentle homogenization in an ice bath. The suspension was centrifuged at 4 °C and 10,000× *g* for 15 min and the supernatant (crude extract) was maintained at 4 °C without appreciable loss of laccase activity for at least 60 days.

### 3.5. Enzymatic Assays

Unless otherwise stated, the laccase activity was determined at 65 °C in McIlvaine buffer [89], pH 4.5, containing 1.0 mmol·L^−1^ 2,2’-azino-*bis*(3-ethylbenzothiazoline-6-sulfonate) (ABTS, Sigma-Aldrich Chem. Co., St. Louis, MO, USA) as substrate in a final volume of 1.0 mL. The reactions were initiated by the addition of the enzyme to the reaction medium and the oxidation of ABTS to ABTS^+^ (ɛ_420, pH 4.5_ = 3.6 × 10^4^ mol·L^−1^·cm^−1^) was monitored continuously under magnetic stirring for 1 min at 420 nm in a Carry 60 UV–Vis spectrophotometer (Agilent Technologies, Santa Clara, CA, USA) equipped with thermostated cell holders. The experimental conditions (reaction times, enzymatic units) employed in all activity measurements were adjusted to guarantee the estimation of initial velocities, and controls without added enzyme were included in each experiment to quantify the non-enzymatic oxidation of the substrate. One enzyme unit was defined as the amount of enzyme that catalyzes the oxidation of 1 µmol ABTS per min. The specific activity was defined as units per milligram total protein. All enzymatic assays were carried out in duplicate.

### 3.6. Purification of Lacps1

*Pycnoporus sanguineus* RP15 was cultivated in 250 mL Erlenmeyer flasks at 70% humidity and under optimized conditions for laccase production (8.0 days, 25 °C, 5.0 g dry wheat bran, 0.8% (*w*/*w*) NH_4_Cl, 50 mmol·L^−1^ CuSO_4_, 18% (*w*/*w*) dry milled corncob, initial moisture of 4.12 mL·g^−1^). The laccase-rich crude extract was adjusted to 60% saturation with solid (NH_4_)_2_SO_4_ under stirring and after standing overnight at 4 °C the precipitate was collected by centrifugation at 10,000× *g* and 4 °C for 15 min, dissolved in a small volume of McIlvaine buffer, pH 4.5, and exhaustively dialyzed against the same buffer. The dialyzed sample was adjusted to 2 mol·L^−1^ (NH_4_)_2_SO_4_ and applied onto a Phenyl-Sepharose CL-4B column (10.0 cm× 2.0 cm) equilibrated and eluted with McIlvaine buffer, pH 4.5, containing 2 mol·L^−1^ (NH_4_)_2_SO_4_, at a flow rate of 120 mL/h. Fractions of 1.0 mL were collected and analyzed for protein (A_280_) and laccase activity. A single activity peak was eluted and the most active fractions were pooled and washed with Millipore Milli Q (EMD Millipore Co., Billerica, MA, USA) ultrapure water in centrifugal concentrators with a 10-kDa cutoff polyethersulfone membrane (Vivaspin 20, GE Healthcare, Buckinghamshire, UK) for salt removal. The purified enzyme (Lacps1) was stored at 4 °C for further use. The purification protocol was repeated 5-fold using five different crude culture extracts from *P. sanguineus*, resulting in five separate preparations of pure Lacps1.

### 3.7. Estimation of Protein and Neutral Carbohydrates 

Protein concentrations were determined as described by Read and Northcote [90] using bovine serum albumin (Sigma-Aldrich Chem. Co., St. Louis, MO, USA) as standard. Total neutral carbohydrates were quantified by the method described by Dubois *et al.* [91], using mannose as standard.

### 3.8. Polyacrylamide Gel Electrophoresis

PAGE was carried out in 7% acrylamide slab gels according to Davis [92]. SDS-PAGE was carried out in 10% acrylamide slab gels as described by Laemmli [93]. The protein bands were revealed using Coomassie Brilliant Blue R-250 (Sigma-Aldrich Chem. Co., St. Louis, MO, USA). The bands with laccase activity after PAGE were visualized by incubating the gel at 65 °C for 30 min in McIlvaine buffer, pH 4.5, containing 1 mmol·L^−1^ ABTS.

### 3.9. Estimation of the Apparent Molecular Mass of the Native Laccase

The apparent molecular mass of Lacps1 in the native state was estimated by high performance liquid chromatography (HPLC) gel filtration, using a Bio-Sil SEC 400 column (Bio-Rad Laboratories, Hercules, CA, USA) according to Souza *et al.* [94]. The molecular mass markers used were bovine myoglobin, ovalbumin and bovine γ-globulin (Sigma-Aldrich Chem. Co. Sigma-Aldrich Chem. Co., St. Louis, MO, USA).

### 3.10. Characterization of the Purified Lacps1 by Mass Spectrometry Analysis

The purified laccase was subjected to SDS-PAGE and the excised band was submitted to *in situ* trypsin digestion followed by MS analysis [94]. The analysis was carried out using an Axima Performance MALDI-TOF/TOF mass spectrometer (Shimadzu-Kratos, Shimadzu Corp., Kyoto, Japan). The peptide mass fingerprint was obtained and the sequences of the tryptic peptides were deduced from a series of *b-* and *y-*ion fragments produced by high-energy collision-induced dissociation (CID-MS/MS). The CID spectra were directly submitted to MASCOT (http://matrixscience.com) against NCBInr database (nonredundant database at the National Center for Biotechnology Information, USA, http://www.ncbi.nlm.nih.gov/).

### 3.11. Effects of Temperature and pH on the Enzymatic Activity of the Purified Lacps1

The optimum pH and temperature for the activity of Lacps1 were determined using RSM. The levels of the independent variables were defined according to a 2^2^ full-factorial CCRD, comprising 11 experimental runs, including 4 axial and 3 central points (triplicate in the central point) (Table 2). The experiment was repeated three times and different pure enzyme preparations were used for each replicate (*n =* 3); the laccase activity was determined in duplicate. The average of the specific laccase activities determined at each pH and temperature condition was used as the response value. The optimization was validated by a triplicate determination of the laccase activity under the optimized conditions.

### 3.12. Thermal and pH Stabilities of the Lacps1

The thermal stability was evaluated by incubating Lacps1 diluted in Millipore Milli Q water for different time intervals at temperatures from 55 to 75 °C. After cooling on ice (1 min), the residual activities were determined at 65 °C, as described above. The pH stability was evaluated by incubating Lacps1 at 4 °C in McIlvaine buffer, pH 2.0–8.0, or glycylglycine buffer, pH 8.5–10.0, for 24 h. The residual activities were estimated in McIlvaine buffer pH 4.5, as described above. The experiments were repeated three-fold with different pure Lacps1preparations (*n =* 3) and the enzymatic assays were performed in duplicate.

### 3.13. Experimental Design, Statistical Analysis and RSM Modeling

The software Statistica, version 11.0 (StatSoft Inc., Tulsa, OK, USA), was employed for the experimental designs (CCRD), and to obtain the ANOVA and the regression coefficients, which were used to derive mathematical models from the experimental data. The variables that exerted significant effects on the response of interest at 95% confidence level (*p* < 0.05) were identified. The quality of fit of the models was expressed by the *R*^2^ coefficients and the statistical significance of the models was checked by the *F*-test using the same software. 

### 3.14. Determination of the Kinetic Parameters and Data Fitting

The values of *V*_M_, *K*_M_ and the Hill coefficient (*n*_H_) for the oxidation of ABTS by Lacps1 at 65 °C were calculated using the SigrafW software, which fits experimental data to the Hill equation using non-linear regression [95]. All experimental kinetic curves were repeated three times, using different pure enzyme preparations, and every experimental point was assayed in duplicate. The kinetic parameters are given as the mean ± SD of the values calculated for three different experiments (*n =* 3). Except for the RSM experiments, data fitting and statistical analyses were carried out using the OriginPro 8 SRO software package (OriginLab Corp., Northampton, MA, USA).

### 3.15. UV–Vis Absorption Spectrum of Lacps1

The absorption spectrum of the enzyme in McIlvaine buffer, pH 4.5, was recorded from 300 to 800 nm, at 25 °C, using a Carry 60 UV–Visible spectrophotometer (Agilent Technologies, Santa Clara, CA, USA) and a 1.0 cm light path quartz cuvette. The experiment was repeated three-fold with different enzyme preparations.

### 3.16. Determination of the Redox Potential of Lacps1

The determination of the *E*° of the Lacps1 T1 center was carried out by following a procedure of protein redox titration employing Fe(dipyridyl)_2_Cl_3_/Fe(dipyridyl)_2_Cl_2_ as mediators [96]. The *E*° for the couple Fe(dipyridyl)_2_Cl_3_/Fe(dipyridyl)_2_Cl_2_ lies at 0.780 V *vs.* NHE [97], which is appropriate for high-redox potential laccases. The components of the redox couple were prepared by mixing FeCl_2_ or FeCl_3_ with 2,2′-dipyridyl in water at a molar ratio of 1:2. As a control, the absorption spectrum of pure Lacps1 (10 mg·mL^−1^ in 0.1 mol·L^−1^ phosphate buffer, pH 6.0) was recorded under anaerobic conditions in the range 550–800 nm, corresponding to the typical absorption band of the T1 copper site. Subsequently, spectra of Lacps1 in the presence of a fixed concentration (0.2 mmol·L^−1^) of the electron donor, Fe(bipyridyl)_2_C1_2_, and increasing concentrations of the electron acceptor Fe(bipyridyl)_2_Cl_3_ (0.01–0.2 mmol·L^−1^) were recorded under the same conditions after reaching the equilibrium, monitoring the concentrations of the oxidized (copper II) and reduced (copper I) states.

The value of the T1 *E*° for Lacps1 was obtained from a Nerst plot of log [(A_0_ − A)/A] *vs.* the potential of the redox couple [Fe(dipyridyl)_2_Cl_3_/Fe(dipyridyl)_2_Cl_2_] *vs.* NHE in Volts, where A_0_ and A corresponded to the absorbances of the solution at 740 nm before and after the reaction equilibrium. The potential of the redox couple was calculated from the Nerst equation associated with the redox species involved (*E* = *E*° + 0.058 log {[Fe(dipyridyl)_2_Cl_3_]/[Fe(dipyridyl)_2_Cl_2_]}), in which the concentrations of each species were obtained from the initial values considering the concentration changes due to the interaction with the enzyme after the equilibrium.

### 3.17. Decolorization of Synthetic Dyes by the Laccase-Rich Crude Extract and Pure Lacps1

Decolorization experiments were performed employing either the laccase-rich crude extract from *P. sanguineus* RP15 or the pure Lacps1 and three different dyes as substrates: the anthraquinone dyes RBBR and RB4, and the triphenylmethane dye BPB (Sigma-Aldrich Chem. Co.). The reaction mixtures contained the dye at final concentrations of 100 mg·L^−1^ (RBBR and RB4) or 25 mg·L^−1^ (BPB) in McIlvaine buffer, pH 4.5, in a final volume of 1 mL. The reactions were performed under magnetic stirring at 25 or 40 °C and initiated by the addition of the enzyme to the reaction media. The enzymatic load was varied from 0.1 to 5.0 U·mL^−1^ and reaction mixtures containing aliquots of boiled crude extract or pure enzyme were used as negative controls. Dye decolorization was followed continuously in a Carry 60 UV–Visible spectrophotometer (Agilent Technologies) equipped with thermostated cell holders by monitoring the decrease of the absorbance at the wavelength of maximum absorbance of each dye (596 nm for RBBR, 592 nm for BPB and 601 nm for RB4). The percentage of decolorization was calculated according to the equation: Decolorization (%) = [A*i* − A*t*/A*i*] × 100, where A*i* is the initial absorbance of the reaction mixture and A*t* is the absorbance after incubation for *t* minutes [83]. The experiments were repeated three times using three different crude extract or pure enzyme preparations for each replicate (*n =* 3). The enzymatic assays were carried out in duplicate.

## Figures and Tables

**Table 1:** Effect of different carbon sources and supplementary carbon and nitrogen sources on the production of laccases by *P. sanguineus* RP15.

Carbon Source		U·g^−1^	U·mL^−1^
Wheat bran		29.8 ± 1.9	5.9 ± 0.4
Steam-exploded sugarcane bagasse		ND ^a^	ND
Raw sugarcane bagasse		ND	ND
Peanut hull		2.1 ± 0.2	0.40 ± 0.03
Rice husk		1.9 ± 0.1	0.40 ± 0.02
Milled corncob		ND	ND
Milled Soybean		ND	ND
Sugarcane trash		ND	ND
**Carbon Supplement (1% *w*/*w*)**		**U·g^−1^**	**%**
None		30.1 ± 1.8	100.0
Peanut hull		31.9 ± 1.4	105.8
Rice husk		29.6 ± 1.1	98.2
Soybean meal		23.7 ± 1.1	78.7
Corn husk		25.5 ± 1.3	84.6
Sugarcane trash		30.5 ± 1.7	101.3
Glucose		28.2 ± 1.1	93.7
Milled corncob		35.5 ± 1.7	117.8
**Nitrogen Supplement**	**% (*w*/*w*)**	**U·g^−1^**	**%**
None	--	33.5 ± 1.7	100.0
Asparagine	1	35.3 ± 1.5	105.6
Casein	1	37.3 ± 1.6	111.2
Soybean meal	1	26.9 ± 1.2	80.4
Glycine	1	29.2 ± 1.3	87.4
Peptone	1	46.0 ± 2.4	137.4
Yeast extract	1	35.8 ± 1.9	107.0
Malt extract	1	39.0 ± 1.9	116.4
NH_4_NO_3_	0.8	31.0 ± 1.7	92.8
KNO_3_	0.8	33.6 ± 1.5	100.5
NaNO_3_	0.8	17.6 ± 0.9	52.2
(NH_4_)_2_SO_4_	0.8	29.4 ± 1.3	87.7
NH_4_Cl	0.8	56.1 ± 2.7	167.8
Urea	0.8	35.9 ± 2.0	107.3

*Pycnoporus sanguineus* RP15 was cultured for 192 h at 25 °C in 5 g dry carbon source (or a mass occupying maximally 1/5 of the total volume of the culture flask) and deionized water (2 mL·g^−1^ dry substrate). Supplementary carbon and nitrogen sources were added to dry wheat bran at the indicated concentrations. The enzymatic assays were performed in duplicate; each experiment was repeated three fold (*n =* 3). Data are presented as means ± SD. ^a^ ND: undetectable by the methods used.

**Table 2:** Experimental conditions and results of the statistical experimental design for the production of laccases by *P. sanguineus* RP15.

Run	Real (Coded) Values				Laccase (U·g^−1^)
	Time	Initial Moisture	Temperature	Milled Corncob	
	(Days)	(mL·g^−1^)	(°C)	(%, *w*/*w*)	
1	7 (−1)	3 (−1)	20 (−1)	10 (−1)	47.5 ± 3.9
2	9 (+1)	3 (−1)	20 (−1)	10 (−1)	56.7 ± 4.1
3	7 (−1)	5 (+1)	20 (−1)	10 (−1)	36.9 ± 5.1
4	9 (+1)	5 (+1)	20 (−1)	10 (−1)	42.9 ± 4.3
5	7 (−1)	3 (−1)	30 (+1)	10 (−1)	49.1 ± 5.7
6	9 (+1)	3 (−1)	30 (+1)	10 (−1)	47.6 ± 5.3
7	7 (−1)	5 (+1)	30 (+1)	10 (−1)	68.2 ± 6.1
8	9 (+1)	5 (+1)	30 (+1)	10 (−1)	59.7 ± 6.3
9	7 (−1)	3 (−1)	20 (−1)	20 (+1)	73.9 ± 6.9
10	9 (+1)	3 (−1)	20 (−1)	20 (+1)	77.2 ± 7.4
11	7 (−1)	5 (+1)	20 (−1)	20 (+1)	45.7 ± 3.9
12	9 (+1)	5 (+1)	20 (−1)	20 (+1)	48.6 ± 4.6
13	7 (−1)	3 (−1)	30 (+1)	20 (+1)	65.7 ± 5.9
14	9 (−1)	3 (−1)	30 (+1)	20 (+1)	74.7 ± 8.4
15	7 (−1)	5 (+1)	30 (+1)	20 (+1)	112.9 ± 10.9
16	9 (+1)	5 (+1)	30 (+1)	20 (+1)	95.1 ± 9.7
17	6 (−2)	4 (0)	25 (0)	15 (0)	115.2 ± 10.6
18	10 (+2)	4 (0)	25 (0)	15 (0)	119.8 ± 12.3
19	8 (0)	2 (−2)	25 (0)	15 (0)	33.1 ± 2.1
20	8 (0)	6 (+2)	25 (0)	15 (0)	78.3 ± 6.4
21	8 (0)	4 (0)	15 (−2)	15 (0)	29.4 ± 2.4
22	8 (0)	4 (0)	35 (+2)	15 (0)	54.6 ± 4.8
23	8 (0)	4 (0)	25 (0)	5 (−2)	51.6 ± 4.1
24	8 (0)	4 (0)	25 (0)	25 (+2)	119.5 ± 12.5
25	8 (0)	4 (0)	25 (0)	15 (0)	136.4 ± 14.7
26	8 (0)	4 (0)	25 (0)	15 (0)	137.2 ± 14.2
27	8 (0)	4 (0)	25 (0)	15 (0)	135.8 ± 13.1

The microorganism was cultured in 5 g wheat bran containing 50 mmol·L^−1^ CuSO_4_ and 0.8% (*w*/*w*) NH_4_Cl. The experiments were conducted in triplicate (*n* = 3) and each enzymatic assay was carried out in duplicate. The average of the laccase production (U·g^−1^) was used as the response value. Data are presented as means ± SD.

**Table 3:** Analysis of variance (ANOVA) for the second-order polynomial models and coefficient values for the production of laccases by *P. sanguineus* RP15.

Effect		Coefficient		*p*-Value	
Intercept		124.33		0.000000	
(x) Time (Q)		−12.54		0.001921	
(y) Initial Moisture (Q)		−23.42		0.000002	
(z) Temperature (L)		9.72		0.011855	
(z) Temperature (Q)		−20.04		0.000014	
(w) Milled Corncob (L)		8.10		0.031957	
Zy		11.25		0.016584	
**Source**	**SS ^a^**	**Df ^b^**	**MSq ^c^**		***F_calc_***
Regression	24,581.59	6	4096.93		13.83
Residual	5923.10	20	296.15		–
Total	30,504.69	26	1173.26		–

*R*^2^ = 0.80; *F* listed 5% = 2.60; ^a^ SS: sum of squares; ^b^ DF: degrees of freedom; ^c^ MSq: mean square.

**Table 4:** Purification of Lacps1.

Step	Total Activity (U)	Total Protein (mg)	Specific Activity (U·mg^−1^)	Yield (%)	Purification (Fold)
Crude extract	548.0	32.70	16.8	100	1
60% (NH_4_)_2_SO_4_	344.7	13.10	26.3	63	1.6
Phenyl-Sepharose	164.4	0.40	411.0	30	24.5

The purification protocol was repeated five-fold. Representative data from one of the purification procedures are presented.

**Table 5:** Experimental conditions and results of the statistical experimental design for the laccase activity.

Run	Real (Coded) Values		U·mg^−1^
	*T* (°C)	pH	
1	70 (−1)	4.0 (−1)	258.4 ± 11.9
2	80 (+1)	4.0 (−1)	176.8 ± 8.5
3	70 (−1)	5.0 (+1)	123.6 ± 6.9
4	80 (+1)	5.0 (+1)	114.9 ± 7.8
5	75 (0)	3.5 (−1.41)	193.8 ± 9.7
6	75 (0)	5.5 (+1.41)	91.4 ± 4.7
7	68 (−1.41)	4.5 (0)	248.0 ± 12.4
8	82 (+1.41)	4.5 (0)	197.9 ± 9.9
9	75 (0)	4.5 (0)	396.1 ± 19.8
10	75 (0)	4.5 (0)	395.4 ± 19.5
11	75 (0)	4.5 (0)	397.1 ± 20.8

The experiment was repeated three times and different pure enzyme preparations were employed for each replicate (*n =* 3). The enzymatic assays were carried out in duplicate, and the response values presented for each run were the means of the three experiments.

**Table 6:** Coefficients values and ANOVA for laccase activity.

Effects		Coefficient		*p-*value	
Intercept		396.25		0.0000002	
pH (L)		−47.99		0.000039	
pH (Q)		−135.57		0.000007	
T (L)		−20.24		0.000224	
T(Q)		−89.72		0.000016	
pH.T		18.23		0.000546	
**Source**	**SS ^a^**	**Df ^b^**	**MSq ^c^**		***F***
Regression	141,950.64	5	28,390.12		–
Residual	106.22	5	21.24		1336.63
Total	142,056.81	10	14,205.68		–

*R*^2^ = 0.99; *F* listed 5% = 5.05; ^a^ SS = sum of squares; ^b^ df = degrees of freedom; ^c^ MSq = mean square.
